# Effects of Magnesium on the Phosphate Toxicity in Chronic Kidney Disease: Time for Intervention Studies

**DOI:** 10.3390/nu9020112

**Published:** 2017-02-06

**Authors:** Yusuke Sakaguchi, Takayuki Hamano, Yoshitaka Isaka

**Affiliations:** 1 Department of Comprehensive Kidney Disease Research, Osaka University Graduate School of Medicine, 2-2, Yamada-oka, Suita 565-0871, Japan; hamatea@kid.med.osaka-u.ac.jp; 2 Department of Nephrology, Osaka University Graduate School of Medicine, 2-2, Yamada-oka, Suita 565-0871, Japan; isaka@kid.med.osaka-u.ac.jp

**Keywords:** magnesium, chronic kidney disease, dialysis, cardiovascular disease, phosphate toxicity, vascular calcification

## Abstract

Magnesium, an essential mineral for human health, plays a pivotal role in the cardiovascular system. Epidemiological studies in the general population have found an association between lower dietary magnesium intake and an elevated risk of cardiovascular events. In addition, magnesium supplementation was shown to improve blood pressure control, insulin sensitivity, and endothelial function. The relationship between magnesium and cardiovascular prognosis among patients with chronic kidney disease (CKD) has been increasingly investigated as it is becoming evident that magnesium can inhibit vascular calcification, a prominent risk of cardiovascular events, which commonly occurs in CKD patients. Cohort studies in patients receiving dialysis have shown a lower serum magnesium level as a significant risk for cardiovascular mortality. Interestingly, the cardiovascular mortality risk associated with hyperphosphatemia is alleviated among those with high serum magnesium levels, consistent with in vitro evidence that magnesium inhibits high-phosphate induced calcification of vascular smooth muscle cells. Furthermore, a harmful effect of high phosphate on the progression of CKD is also attenuated among those with high serum magnesium levels. The potential usefulness of magnesium as a remedy for phosphate toxicity should be further explored by future intervention studies.

## 1. Introduction

Magnesium is an essential mineral for human health and acts as a co-factor for more than 500 enzymatic reactions in the body. Dietary magnesium intake in developed countries has decreased over the past decades owing to the increased consumption of low-magnesium diets, such as processed foods and fast foods; as a result, more than half of the US population do not meet the estimated average requirement of dietary intake of magnesium [1]. Meta-analyses of epidemiological studies in the general population have linked the lower dietary magnesium intake to an increased risk of cardiovascular diseases [2,3,4] and metabolic syndrome [5,6,7]. In addition, double-blind randomized controlled trials have demonstrated that magnesium supplementation improves blood pressure control [8], insulin sensitivity [9], and endothelial function [10,11]. Consistently, a number of experimental studies have demonstrated that magnesium is protective against endothelial cell damage and oxidative stress [12].

Despite this evidence implicating magnesium as a protective mineral for the cardiovascular system, this divalent cation has received little attention in the field of chronic kidney disease (CKD). However, the low magnesium status may also be unfavorable for CKD patients since it is associated with inflammation, atherosclerosis, and intradialytic hypotension among patients undergoing hemodialysis [13,14]. More importantly, given the huge impact of the dysregulated mineral and bone metabolism on cardiovascular risk of patients with CKD, where calcium, phosphate, and parathyroid hormone have been regarded as central players, magnesium may also serve a unique function. For example, magnesium is known to inhibit crystallization of calcium phosphate. Magnesium can also suppress parathyroid hormone secretion by acting on the calcium-sensing receptors on the parathyroid glands [15,16]. In this review, we will summarize the recent advances of magnesium research in CKD with a particular focus on vascular calcification and phosphate toxicity.

## 2. Magnesium and Vascular Calcification

### 2.1. Clinical Studies

Vascular calcification is one of the most important aspects of the mineral and bone disorders of CKD. In particular, coronary artery calcification strongly predicts an incidence of cardiac events and mortality in patients with CKD [17,18,19,20,21,22,23]. Although a therapeutic strategy for vascular calcification is not well established, magnesium has long been presumed to have a pathophysiological relevance in extraosseous mineralization since it can inhibit, at least chemically, the formation and growth of calcium-phosphate crystals–hydroxyapatite [24]. Meema et al. were the first to suggest the clinical linkage between magnesium and vascular calcification [25]. They examined the longitudinal relationship between serum magnesium levels and peripheral arterial calcifications of 44 patients receiving peritoneal dialysis with a median follow-up period of 27 months. They found that a lower serum magnesium level was closely associated with the progression of calcification. This historical finding proposed a hypothesis that mild hypermagnesemia in uremic patients is beneficial as it can alleviate the progression of vascular calcification. Later, cross-sectional studies of hemodialysis patients have confirmed the significant association of lower serum magnesium levels with the presence of calcification of the hand arteries [26] and mitral annular calcification [27]. We recently analyzed the density of coronary artery calcification of 109 non-dialysis CKD patients mainly with type 2 diabetes mellitus and showed that the density increased as serum magnesium levels became lower [28]. This relationship was particularly pronounced among those with higher serum phosphate levels, implying that magnesium is more likely to be involved in a denser calcified lesions, i.e., media calcification, induced by phosphate.

Spiegel et al. have reported that magnesium supplementation for hemodialysis patients may be useful to suppress the progression of coronary artery calcification in a small-scale uncontrolled trial [29]. Recently, Tzanakis et al. conducted a pilot intervention trial of 59 hemodialysis patients who were randomly assigned to a 12-month treatment of either magnesium-containing phosphate binders (magnesium carbonate/calcium acetate) or calcium-containing phosphate binders (calcium acetate) [30]. The two groups were then compared with respect to the progression of arterial calcification. At the end of the study, the patients in the magnesium-containing phosphate binder group were more likely to show a regression of arterial calcification than those in the calcium-containing phosphate binder group. While this study proposes a promising therapeutic effect of magnesium binders on vascular calcification, the sample size was relatively small and the evaluation methodology of vascular calcification might be problematic in accuracy and reproducibility. Further large-scale trials with a more validated measure of calcification (e.g., coronary artery calcification score) are required.

### 2.2. Experimental Studies

Animal studies, both in CKD and non-CKD models, suggested that magnesium inhibits vascular calcification in vivo although it has long been unclear whether magnesium directly acts on calcification [31,32]. Recently, the underlying mechanisms by which magnesium inhibits vascular calcification have been intensely investigated. Montezano et al. firstly reported an inhibitory effect of magnesium on the process of calcification in vitro [33]. They found that magnesium effectively prevents calcification and osteogenic transformation of rat vascular smooth muscle cells (VSMCs) induced by phosphate overload. After this report, similar results have repeatedly been shown in in vitro and ex vivo experiments by many researchers [34,35,36,37,38,39,40,41]. Although the exact pathways by which magnesium alleviates phosphate-induced calcification of VSMCs are not well understood, Louvet et al. negated the previously postulated hypothesis that magnesium alters the physicochemical nature of calcium phosphate crystal composition or structure based on their observation using micro-Fourier transform infrared spectroscopy, field effect scanning electron microscopy, and energy dispersive X-ray spectrometry [41]. Interestingly, the anti-calcification property of magnesium was largely abolished after a pharmacological inhibition and/or siRNA knockdown of TRPM7 (transient receptor potential melastatin 7), a ubiquitous cell membrane transporter of magnesium, implicating an intracellular role of magnesium in its anti-calcification process [33,37]. In this regard, Montes de Oca et al. showed that magnesium suppressed the nuclear accumulation of β-catenin and its downstream genes expression in human VSMCs induced by phosphate overload [37]. Notably, the suppressive effect of magnesium on the Wnt/β-catenin pathway was again abrogated by the inhibition of TRPM7. Further studies should elucidate how magnesium directly interacts with this pathway. On the other hand, Louvet et al. reported an involvement of several kinds of microRNAs (miR-30b/miR-133a/miR-143) expressed in VSMCs that negatively regulate osteogenic gene expressions [41]. They found that the downregulation of these microRNAs induced by phosphate overload was restored by increasing the medium concentration of magnesium, although the mechanism by which magnesium alters the expression of these microRNAs is yet to be known.

The inhibitory effect of magnesium on vascular calcification may also be attributed from its function in the extracellular space. In the circulation, where calcium and phosphate are supersaturated, some kinds of proteins, such as Fetuin-A, interact with a calcium ion on the surface of the calcium phosphate cluster to form calciprotein particles (CPPs) that have lower cytotoxicity compared with a naked calcium-phosphate crystal [42]. CPPs are primarily formed as a sub-nanometer-sized spherical particle called primary CPPs, which contains amorphous calcium phosphate. Meanwhile, primary CPPs aggregate and undergo spontaneous rearrangement to a larger needle-shaped particle called secondary CPPs [43]. Aghagolzadeh et al. have recently shown that secondary CPPs, but not primary CPPs, have a pronounced potential to induce calcification [44]. It is important to note that magnesium suppresses the maturation of CPPs in vitro [45]. Consistently, in a clinical setting, serum magnesium level is shown to be one of the major determinants of CPP maturation time (T_50_) in serum, a strong predictor of mortality in patients with kidney disease [46]. In this regard, the serum magnesium level is thought to be clinically relevant despite the fact that it does not reflect well the total body magnesium. Taken together, the anti-calcification property of magnesium may have partly originated from its extracellular function of inhibiting CPPs maturation.

## 3. Magnesium and Clinical Outcomes in CKD

### 3.1. Magnesium and Cardiovascular Outcomes in CKD

Based on the evidence discussed above, it is anticipated that magnesium is protective against cardiovascular risk of patients with CKD. To test this hypothesis, we examined the relationship between serum magnesium levels and cardiovascular mortality risk in a cohort of 142,555 patients undergoing hemodialysis with a one-year follow-up period [47]. As expected, lower serum magnesium levels were associated with an increased risk of all-cause and cardiovascular death after adjustment for various clinical factors such as nutritional status, inflammation, and other MBD-related factors. We also found an inverse association between serum magnesium levels and parathyroid hormone levels which is in consistent with in vitro evidence [16]. The association between serum magnesium levels and mortality has been confirmed among incident hemodialysis patients, peritoneal dialysis patients, and non-dialysis CKD patients with a longer follow-up period [48,49,50,51,52,53,54,55,56,57]. Two of these studies have shown an association between serum magnesium levels and the risk of sudden death [51,52]. Importantly, our study also indicated that the risk of death was gradually increased when pre-dialysis serum magnesium levels exceeded 3.0 mg/dL. Similar J-shaped association was also shown in a cohort of hemodialysis patients with secondary hyperparathyroidism [53]. Although the reason underlying this unexpected finding is uncertain, excess magnesium may be unfavorable, for example, for electrophysiological activity of myocardium. Therefore, it is of importance to determine not only the lower limit, but also the higher limit of the target range of magnesium by future intervention studies.

**Figure 1:**
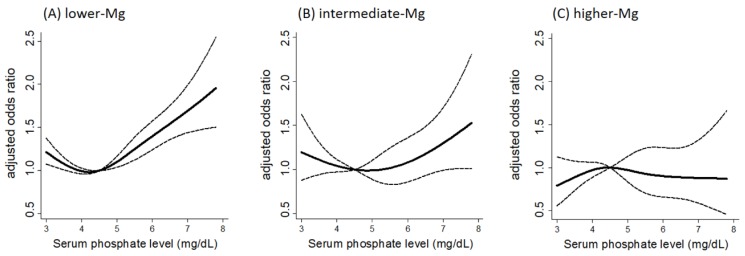
Interaction between serum magnesium and phosphate levels on the risk of cardiovascular death among patients undergoing hemodialysis. Adjusted odds ratio for cardiovascular mortality among patients with serum magnesium levels of (**A**) <2.7 mg/dL; (**B**) ≥2.7, <3.1 mg/dL; and (**C**) ≥3.1 mg/dL. The dashed line represents the 95% confidence interval. The reference serum phosphate value is at 4.5 mg/dL. The *p*-value for interaction (serum magnesium vs. serum phosphate) on the risk of cardiovascular death is 0.03 (cited from [58]).

Given the in vitro evidence showing the potent protective effect of magnesium against phosphate-induced vascular calcification, it can be assumed that the influence of magnesium on the cardiovascular risk is particularly manifested among patients with high phosphate levels. In fact, there is a significant interaction between serum levels of magnesium and phosphate on the cardiovascular mortality risk of hemodialysis patients; the risk associated with hyperphosphatemia was aggravated in those with lower serum magnesium levels, whereas the risk was substantially attenuated in those with higher serum magnesium levels [58] (Figure 1). This result proposes a new therapeutic concept that increasing magnesium levels could attenuate the cardiovascular risk derived from hyperphosphatemia.

### 3.2. Magnesium and Phosphate Balance in the Risk of Progression of CKD

The counteracting action of magnesium against the phosphate-induced pathological condition, as evident in vascular calcification, might also be exerted to other target organs of phosphate toxicity. It has long been known that excess phosphate causes tubular injury and interstitial fibrosis in animal models of CKD [59,60,61,62,63]. Although one study has reported a neutral effect of hyperphosphatemia on the risk for progression of CKD [64], most of the previous cohort studies and their meta-analysis have shown a link between high serum phosphate levels and an increased risk of CKD progression [65,66,67,68,69,70,71,72,73,74]. Therefore, the kidney is considered as one of the representative targets of phosphate toxicity. On the other hand, recent observational studies have advocated a link between magnesium and the progression of CKD. An inverse association between serum magnesium levels and the risk of deterioration of kidney function and/or end stage kidney disease has been reported in a cohort of CKD [75,76] as well as in the general population [77]. Lower dietary magnesium intake is also associated with a rapid decline in kidney function in a population-based cohort of estimated glomerular filtration rate (eGFR) ≥ 60 mL/min/1.73 m^2^ [78]. While several underlying mechanisms are postulated, it might be possible that magnesium particularly mitigates the kidney damage induced by phosphate overload. This notion is supported by a retrospective cohort study of 311 non-diabetic CKD patients with a median follow-up of 44 months that showed the patients with lower serum magnesium–higher serum phosphate were at a 2.07-fold higher risk for end-stage kidney disease when compared with those with higher serum magnesium–higher serum phosphate whose risks were equivalent to patients with lower serum phosphate levels [79]. Consistently, an in vitro experiment revealed that a proximal tubular cell injury caused by high phosphate was attenuated by increasing medium magnesium concentration partly through a restoration of mitochondrial membrane potential [79]. This evidence suggests that the concept of magnesium as an inhibitor of phosphate toxicity may be applicable to not only the cardiovascular risk but also the risk of CKD progression.

## 4. Low Dietary Magnesium Intake of Hemodialysis Patients

### 4.1. Causes of Magnesium Deficiency

The magnesium content in the body is tightly regulated by an orchestrated interaction between the intestines, kidneys, and bones [80]. Magnesium deficiency, therefore, is mainly caused by (1) low magnesium intake; (2) reduced gastrointestinal absorption; and (3) enhanced urinary excretion. 

(1) Low Magnesium Intake

Magnesium is abundantly found in green leafy vegetables, seaweeds, beans, legumes, and nuts. Processed foods and fast foods are poor in magnesium. Although severe magnesium deficiency due to low dietary magnesium intake is uncommon, except for patients who are maintained on magnesium-deficient parenteral nutrition, mild deficiency is becoming prevalent as the consumption of processed foods and fast foods has increased, especially in developed countries [1]. 

(2) Reduced Gastrointestinal Absorption

Intestinal absorption of magnesium mainly occurs in the jejunum, ileum, and colon through both an active transcellular transportation and passive paracellular diffusion. When magnesium intake is low, the active transport pathway via TRPM6 becomes dominant. Loss-of-function mutations of TRPM6 is a cause of autosomal recessive familial hypomagnesemia with secondary hypocalcemia. Recently, it has become widely recognized that use of proton pump inhibitors induces hypomagnesemia even in hemodialysis patients [81]; the underlying mechanism is supposed to be an impairment of magnesium absorption in the colon, possibly through a PPI-induced elevation of luminal pH, which inactivates TRPM6.

(3) Enhanced Urinary Excretion

About 70% of magnesium in the plasma is filtered by the glomeruli. Filtered magnesium is reabsorbed in the proximal tubules (10%–25%), the loop of Henle (50%–70%), and distal convoluted tubules (~10%) where fine-tuning of the total magnesium reabsorption takes place by TRPM6. Fractional excretion of magnesium (FEMg) can be as low as 0.5%. On the other hand, FEMg of more than 4% in the presence of hypomagnesemia is considered as renal magnesium wasting. In addition to genetic disorders, there are many clinical factors that cause renal magnesium wasting. Among them, the most frequent cause is drug-induced hypomagnesemia (e.g., diuretics, calcineurin inhibitors, cisplatin, aminoglycosides, and epidermal growth factor receptor inhibitors (cetuximab), etc.). Insulin resistance is another important cause of magnesium loss from the kidney and it appears that a resultant magnesium deficiency aggravates the glucose metabolism [82]. 

### 4.2. Imbalance between Magnesium and Phosphate in Hemodialysis Patients

Since urinary magnesium excretion is mostly negligible among hemodialysis patients, serum magnesium levels of these patients are largely determined by dietary magnesium intake as well as dialysate magnesium concentrations. Under the same dialysate magnesium concentration, serum magnesium levels are well correlated with the dietary amount of magnesium [83]. Luis et al. reported that the daily amount of magnesium intake of hemodialysis patients is very low, and only 2% of patients consume magnesium above the minimum requirement [84]. This is probably because magnesium-rich foods are also rich in potassium, which should be restricted for patients with end-stage kidney disease. More importantly, processed foods are deficient in magnesium since food-processing causes a substantial loss of magnesium. These foods, at the same time, contain a large amount of inorganic phosphate as food additives. Therefore, it is anticipated that the processed foods not only decrease the consumption of magnesium but also enhance that of phosphate, inducing the “high phosphate-low magnesium” condition. Future studies should examine the influence of the dietary imbalance of magnesium and phosphate on the cardiovascular risk of dialysis patients.

## 5. How Do We Increase the Magnesium Status of Dialysis Patients?

There are several ways to elevate serum magnesium levels of patients receiving hemodialysis. While it would be unrealistic in clinical practice for dialysis patients to increase their dietary magnesium intake since they need to restrict potassium, magnesium supplementation can increase serum magnesium levels without affecting potassium levels. In this regard, magnesium-containing phosphate binders are useful and can correct the low magnesium–high phosphate status. It has been reported that the magnesium-containing phosphate binder suppresses vascular calcification more effectively than other types of binders in an animal model of CKD [85] as well as hemodialysis patients [30]. In addition, this kind of binder has several advantages, such as being inexpensive and having fewer gastrointestinal side-effects [86]. Whether the magnesium-containing binder is superior to other types of binders in terms of hard outcomes must be further investigated.

Another expeditious and secure method to control magnesium levels is to alter dialysate magnesium concentrations. Notably, dialysate magnesium concentrations may affect intradialytic hemodynamics since magnesium directly influences cardiac contractility and vascular tone. It has been reported that the use of a low magnesium–low calcium dialysate increases the occurrence of intradialytic hypotension and decreases cardiac outputs [14]. Therefore, a higher magnesium dialysate may be beneficial for dialysis patients not only by increasing serum magnesium levels but also by stabilizing intradialytic hemodynamics.

## 6. Conclusions

In the current clinical practice of dialysis patients, the primary treatment option for hyperphosphatemia is to decrease the phosphate load by dietary management and the use of phosphate binders, as well as to remove phosphate by dialysis therapy. Despite the integrative approach by these therapeutic options, however, the problems related to hyperphosphatemia have not been fully resolved over the past decades because of poor adherence to the treatment, side-effects of the drugs, and nutritional disadvantages inherent to dietary restriction. We may have to recognize that the current strategy to reduce phosphate load is reaching a limit and develop an additional way to alleviate the harmful effects of phosphate. Although more compelling data are needed to justify the use of magnesium as a “remedy for phosphate toxicity” in clinical practice, it is worth testing whether the correction of low magnesium status improves the prognosis of patients with hyperphosphatemia. At the same time, it is important to clarify to what degree serum magnesium levels can be safely elevated since excessively high serum magnesium levels may also be harmful [47]. Further efforts are clearly needed to establish the clinical significance of magnesium among patients with CKD.
